# Gelation during Ring-Opening Reactions of Cellulosics with Cyclic Anhydrides: Phenomena and Mechanisms

**DOI:** 10.1021/acs.biomac.4c01081

**Published:** 2024-11-21

**Authors:** Stella P. Petrova, Zhaoxi Zheng, Daniel Alves Heinze, Valerie Vaissier Welborn, Michael J. Bortner, Klaus Schmidt-Rohr, Kevin J. Edgar

**Affiliations:** † Department of Chemistry, Virginia Tech, Blacksburg, Virginia 24061, United States; ‡ Department of Sustainable Biomaterials, Virginia Tech, Blacksburg, Virginia 24061, United States; § Macromolecules Innovation Institute, Virginia Tech, Blacksburg, Virginia 24061, United States; ∥ Department of Chemistry, Brandeis University, Waltham, Massachusetts 02453, United States; ⊥ Department of Chemical Engineering, Virginia Tech, Blacksburg, Virginia 24061, United States

## Abstract

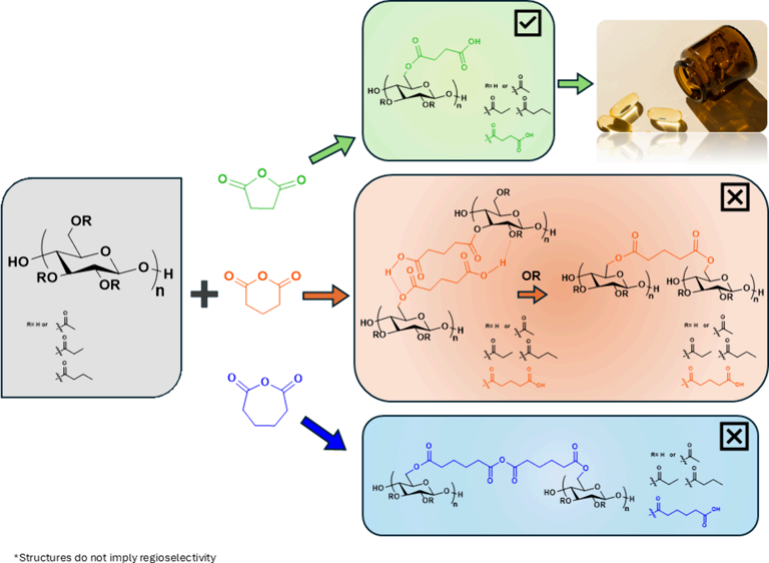

Cellulose esters are used in Food and Drug Administration-approved oral formulations, including in amorphous solid dispersions (ASDs). Some bear substituents with terminal carboxyl moieties (e.g., hydroxypropyl methyl cellulose acetate succinate (HPMCAS)); these ω-carboxy ester substituents enhance interactions with drug molecules in solid and solution phases and enable pH-responsive drug release. However, the synthesis of carboxyl-pendent cellulose esters is challenging, partly due to competing reactions between introduced carboxyl groups and residual hydroxyls on different chains, forming either physically or covalently cross-linked systems. As we explored ring-opening reactions of cyclic anhydrides with cellulose and its esters to prepare polymers designed for high ASD performance, we became concerned upon encountering gelation. Herein, we probe the complexity of such ring-opening reactions in detail, for the first time, utilizing rheometry and solid-state ^13^C NMR spectroscopy. Gelation in these ring-opening reactions was caused predominantly by physical interactions, progressing in some cases to covalent cross-links over time.

## 1 Introduction

The biopolymer cellulose is important in its natural role in reinforcing plant cell walls, and in commercial products such as paper and cotton, while its esters are critical elements of applications including renewable plastics and packaging, food thickeners, displays, electronics, and biomedicine.^1−3^ Cellulose esters (e.g., HPMCAS and cellulose acetate phthalate) have been important biobased polymers for improving the performance of pharmaceutical formulations, for example, as enteric coatings for drug delivery vehicles.^4,5^ They are highly favored as a class for such applications because of their beneficial properties, including their generally benign nature, sustainable sourcing, good film-forming properties (for dosage coating), and high glass transition temperatures.^6^

The Edgar group has designed novel cellulose ω-carboxyalkanoate derivatives modified with succinate, glutarate, and adipate substituents for use as ASD polymers.^7−9^ ASDs have become important in the last two decades in oral drug delivery and comprise an amorphous active pharmaceutical compound molecularly dispersed within an amorphous polymeric substrate, thereby improving drug dissolution through maintaining the drug in a high energy state (its amorphous form), which is thermodynamically unstable relative to its crystalline form. The drug dissolves from the ASD to create a supersaturated solution (drug concentration that exceeds its thermodynamic solubility but not its (higher) amorphous solubility), creating a greater chemical potential difference across the epithelium, simultaneously enhancing both solubility and diffusion. Therefore, ASDs address both impediments (solubility and permeation) to drug bioavailability defined in the Biopharmaceutical Classification System.^10,11^

Recently, the Edgar laboratory demonstrated a scalable, one-pot, efficient approach to creating a library of cellulose ester polymers designed for ASD performance, through the ring-opening of succinic (SA) or glutaric anhydrides (GA) with microcrystalline cellulose or cellulose ester derivatives such as acetate, acetate propionate, or acetate butyrate for ASD structure–property performance studies.^9^ A DMAP/pyridine catalyst system facilitated ring-opening esterification between cellulosic hydroxy groups and succinic or glutaric anhydride while avoiding the cross-linking that is inevitable when carrying out such reactions under acid catalysis.^12,13^ We were surprised in that work to observe gel formation during these reactions even though succinic and glutaric anhydrides have more stable ring sizes (5 and 6, respectively) than the more reactive adipic anhydride (7) studied previously.^14,15^ Gelation was especially prominent when reacting microcrystalline cellulose with GA, consistently occurring within 2–24 h. Furthermore, we also observed gelation regularly in reactions between cellulose ester substrates containing a high starting degree of substitution of OH groups (DS(OH)) and glutaric anhydride, often impeding high conversion to the desired product.^9^ The degree of substitution (DS) is defined as the average number of substituted groups per anhydroglucose unit. While gelation phenomena have been observed before in reactions of cellulose with SA and AA, in-depth mechanistic exploration has not been reported.^3,15,16^

**Figure 1:**
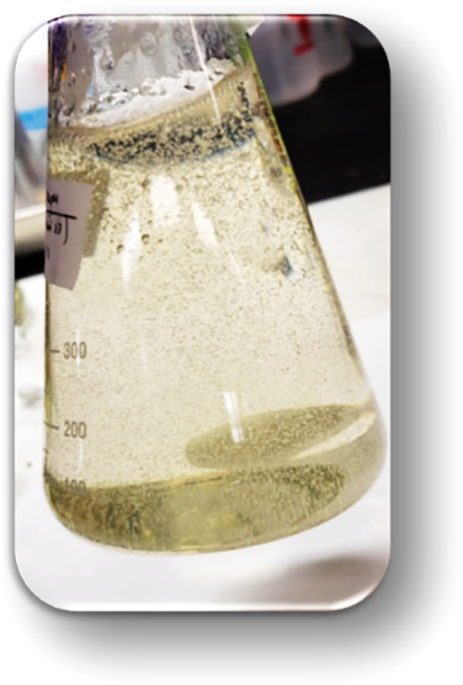
Image of physically gelled cellulose glutarate.

Cellulosic-based gels have been reported to form by physical associations or by covalent chemical linkages (Figure 1). For example, investigation of thermally induced microcrystalline cellulose gelation in aqueous NaOH and thiourea revealed that as temperature increased, the polymer began to self-associate through interactions of hydroxy groups on different cellulosic chains, leading to physically cross-linked networks, aggregation, and subsequent gelation.^17^ Weng et al. did not observe any crystalline regions by wide-angle X-ray scattering (WAXS), indicating that gelation resulted from random hydrogen bonding (H-bonding) between various OH groups rather than the formation of crystalline order. On the other hand, there are reports of covalent cross-linking of cellulose by multifunctional cross-linkers such as dialdehydes, epichlorohydrin, aldehyde-acids, and polyfunctional carboxylic acids such as citric acid and, of particular interest for the present work, *glutaric acid*.^18^

In our previous exploration of the esterification of cellulose and its derivatives using adipic anhydride (AA), we learned that reactions with the rather reactive 7-membered anhydride were exceptionally prone to cross-linking and gelation. After AA was stored for only days or weeks, homopolymerization ensued to form poly(adipic anhydride) or AA oligomers. If oligomeric or polymeric impurities were present in the AA reagent, gelation occurred quickly upon addition to the cellulose reaction mixture.^14,15^ Even when freshly purified AA was added immediately to the reaction vessel, thermally driven ring-opening and subsequent AA homopolymerization occurred in some cases, affording cross-linked products.^15^ FTIR measurements of the cross-linked material did show resonances corresponding to anhydride linkages, indicating that AA homopolymerization, either self-initiated or initiated by a cellulose hydroxy group, was followed by reaction of the poly(anhydride) with OH groups on a nearby cellulose chain (reaction at the anhydride carbonyl proximal to the initiating cellulose chain results in a cross-link).^14^ One might predict that ring opening by weakly nucleophilic carboxylate groups should be slow; however, poly(adipic anhydride) (pAA) was consistently observed in these reactions, even in products where reaction conditions were very carefully controlled and pure (within ^1^H NMR detection limits) AA was added. Some reports have indicated that smaller cyclic anhydrides such as SA can also cross-link polysaccharides.^19−21^ The handful of reports on cellulose esterification with GA (6-membered ring) do not mention gelation except for manuscripts where gelation was intentional to produce hydrogels.^3,16,22^

Reaction of polysaccharides with epoxides in aqueous alkaline media results in substitution with oligo(hydroxyalkyl) groups, since the terminal alkoxylalkyl moiety has wider approach angles than do anhydroglucose hydroxy groups. We postulated that, by analogy, gelation in esterification with cyclic anhydrides is caused by anhydride (e.g., GA) oligomerization from the cellulose hydroxy and/or hydroxyalkyl groups, creating oligo(anhydride) chains that could react with neighboring hydroxy groups present on other cellulosic molecules, thereby leading to cross-linking and gelation if the reaction occurred at the anhydride carbonyl proximal to a cellulose chain. Our alternative hypothesis proposed that gelation in such reactions was instead caused by physical interactions. Rheometry was used to probe the viscoelastic behavior of gelled samples, which should be sensitive to the type of cross-linking (physical or covalent). To quantify and further evaluate whether cross-links were covalent or physical, we analyzed gelled and nongelled materials by solid-state NMR spectroscopy, looking for tell-tale resonances that would be specific to the hypothesized covalent cross-links. We sought overall to provide a clear description of the gelation phenomena in reactions of polysaccharides with cyclic anhydrides, give insight into their scope and potential prevention, and confirm or refute our hypotheses with regard to the cross-linking mechanisms.

## 2 Materials and Methods

### 2.1 Materials

Avicel microcrystalline cellulose (MCC_3.00_, *M*_n_ ∼ 8 kDa, as reported by detailed solid-state NMR analysis,^23^ or *M*_n_ ∼ 36 kDa reported by the manufacturer) was dried at 50 °C under vacuum overnight prior to use. Herein subscripts next to each acronym indicate the DS(OH) available for esterification. 4-Dimethylaminopyridine (DMAP) (Sigma-Aldrich, 99%), 1,3-dimethyl-2-imidazolidinone (DMI, TCI, >99.0%), *N*,*N*-dimethylacetamide (DMAc, anhydrous, Sigma-Aldrich, ≥99.8%), pyridine (99+%, extra dry, ACROS organics), LiCl (Fisher Scientific), molecular sieves (Grade 514, 4 Å, Fisher Scientific), glutaric anhydride (GA, 99.9% pure, anhydrous, CHEM-IMPEX Inc., stored in a desiccator under vacuum), acetone (Fisher Scientific), diethyl ether (Sigma-Aldrich), and methanol (Fisher Scientific) were used as received. Regenerated cellulose dialysis membrane (Spectra/Por, 3.5 kDa MWCO) was kept at 4 °C, and DMI and DMAc were kept over 4 Å molecular sieves under N_2_ atmosphere. HCl (0.1 N) was made by diluting stock 5 N HCl with deionized water (diH_2_O) to the desired concentration.

### 2.2 NMR Measurements

All solid-state NMR experiments were performed on a Bruker Avance Neo 400WB NMR spectrometer using a Bruker double-resonance 4 mm magic-angle spinning (MAS) probe operating at 400 and 100 MHz for ^1^H and ^13^C, respectively; details are given below. All solid-state NMR samples were purified through extensive washing with organic solvent (acetone or methanol) and/or through dialysis against diH_2_O for several days (see Methods). All purified samples were also freeze-dried prior to analysis, ensuring that products were solid and dry. Solution state ^1^H NMR spectra were obtained on a Bruker Avance II 500 MHz spectrometer in D_2_O at room temperature (RT) with 64 scans. Chemical shifts were reported relative to the D_2_O solvent peaks at 4.9 ppm.

### 2.3 Synthesis of the Noncross-Linked Polymer Sample (MCC_3.00_-GA1)

Microcrystalline cellulose (1.00 g, 6.17 mmol) was dried overnight under vacuum at 50 °C, then dissolved in DMAc/5.0% w/v LiCl (80 mL) according to a previous protocol and allowed to stir overnight at RT.^24^ Then DMAP (0.102 g, 0.835 mmol) dissolved in anhydrous pyridine (1.0 mL, 12.41 mmol) was added to the cellulose solution at RT, and the oil bath temperature was increased to 80 °C. GA (0.826 g, 7.24 mmol) was dissolved in DMAc (2.5 mL) and added to the reaction mixture dropwise under N_2_ at 80 °C. The solution was stirred for 12 h, then cooled to RT. Lumps of gelled material were observed in the crude polymer solution. This mixture was placed in dialysis tubing directly and then dialyzed against diH_2_O for 7 days. Then, the dialyzed polymer solution was placed in a 50 mL conical tube. Since gel flakes were present, the conical tube was centrifuged at 10,000 rpm for 45 min to spin the gel material to the bottom of the tube. Then, the liquid fraction was decanted and placed in dialysis tubing to continue dialyzing against diH_2_O for 3 d. After 3 days, the liquid fraction was frozen, then freeze-dried. In this product, carboxylates were intentionally not neutralized, affording a white, spongy material. When the freeze-dried polymer product was placed in phosphate-buffered saline (PBS) at pH 6.8, it dissolved almost immediately upon vortexing. This sample (MCC_3.00_-GA1 or “non-gelled”) was subjected to solution-state and solid-state NMR analysis.

### 2.4 Synthesis of Cross-Linked Sample MCC_3.00_-GA2

Microcrystalline cellulose (1.00 g, 6.17 mmol of repeat units) was dried overnight under vacuum at 50 °C, then dissolved in DMAc/5.0% w/v LiCl (45 mL) according to a previous protocol.^24^ DMAP (0.105 g, 0.859 mmol) was weighed and added to anhydrous pyridine (1.0 mL, 12.41 mmol); then this solution was added to the cellulose solution at RT. The oil bath temperature was increased to 80 °C, and GA (0.827 g, 7.25 mmol) dissolved in DMAc (2.5 mL) was added dropwise at 80 °C. The solution was stirred for 3 days at 80 °C under N_2_. The cooled solution was added to 0.8 L of acetone with vigorous stirring to precipitate the product. The reaction solution was very viscous and contained lumps of gel-like material as it was precipitated. The solid product was isolated by vacuum filtration and then washed with another 1 L of acetone. Then, the product was redissolved in diH_2_O (0.3 L); however, insoluble gelled globules were still observed in the solution. The gelled material was allowed to settle to the bottom of the beaker and the water layer was decanted, and then placed in dialysis tubing (ionized cellulose glutarate is soluble in water). The settled gel fraction was placed in a separate dialysis tube. Each dialysis tube was placed in its own beaker and dialyzed against diH_2_O for 5 days. Then the dialysis tube containing the decanted liquid fraction was dialyzed against 1 L of 0.1 N HCl for 24 h to protonate and reprecipitate the polymer inside the tube. This precipitated polymer was dialyzed against diH_2_O again (2 days) to remove residual HCl, frozen, and freeze-dried. However, when the freeze-dried polymer was placed in PBS at pH 6.8 to ionize the carboxylates and solubilize the polymer, the product swelled only in the PBS and did not dissolve. Therefore, we speculated that this polymer had at least partially cross-linked. The lyophilized sample (MCC_3.00_-GA2) was subjected to solid-state NMR analysis to evaluate whether cross-linking was physical or covalent.

### 2.5 Synthesis of Cross-Linked Polymer Samples MCC_3.00_-GA3 and MCC_3.00_-GA3a

Microcrystalline cellulose (1.00 g, 6.17 mmol) was dried overnight under vacuum at 50 °C, then dissolved in DMAc/5.0% w/v LiCl (50 mL) according to a previous protocol and stirred overnight at RT.^24^ DMAP (0.100 g, 0.819 mmol) dissolved in anhydrous pyridine (1.0 mL, 12.41 mmol) was added to the cellulose solution at RT, and the temperature was increased to 80 °C. A solution of GA (0.827 g, 7.25 mmol) in DMAc (2.5 mL) was added dropwise under N_2_. The mixture gelled overnight and was cooled for approximately 12 h. The crude reaction mixture was split into two portions. Part 1, which we will refer to as MCC_3.00_-GA3, was purified and processed immediately; it was placed directly into 0.3 L of MeOH, stirred, filtered, and the precipitate reslurried in MeOH three times, yielding gelatin-like chunks in the methanol. The chunks were then placed in a beaker containing diH_2_O (0.25 L). The material swelled significantly, absorbing all the water, so an additional 0.25 L (0.5 L total) of water was added. The remaining crude sample, which we will refer to as MCC_3.00_-GA3a, was stored in a glass jar and kept unprocessed and sealed for 1 week at RT. After 1 week, both MCC_3.00_-GA3 (processed polymer) and MCC_3.00_-GA3a (unprocessed polymer) were analyzed rheometrically. Then, MCC_3.00_-GA3a was purified by dialyzing against MeOH for 3 days, then diH_2_O for 5 days prior to freeze-drying. The lyophilized MCC_3.00_-GA3a polymer was further analyzed by solid-state NMR.

### 2.6 Rheological Characterization of Cross-Linked MCC_3.00_-GA3 and MCC_3.00_-GA3a

The rheological behavior of gelled samples was evaluated at 22 °C with a Discovery HR-2 instrument using a 25 mm parallel plate geometry and a Peltier plate with a 0.5 mm gap. Briefly, crude, gelled material was placed between the plates and equilibrated for 30 s prior to testing. Complex viscosity was obtained from an amplitude sweep at 10.0 rad/s and with strain varying logarithmically from 0.1 to 1000.0% and then back from 1000.0 to 0.1%. The difference in the magnitude of the complex viscosity between testing from low to high strains and then from high to low strains was used to confirm hysteresis resulting from physical bond breakage due to the imposed strain. Data were exported using TRIOS Software.

### 2.7 Solid-State NMR Analysis of Cross-Linked MCC_3.00_-GA Samples

For solid-state NMR analysis with MAS at 14 kHz on a Bruker Avance Neo 400WB NMR spectrometer at a 100 MHz ^13^C resonance frequency, lyophilized dry samples were center-packed into 4 mm zirconia rotors with Kel-F caps. Nearly quantitative ^13^C NMR spectra were measured by multiple cross-polarization (multiCP)^25,26^ NMR with recycle delays of 3 s for both MCC_3.00_-GA1 and -GA3a and 7 s for MCC_3.00_-GA2, and 4 repolarization delays of 1.5 and 3.5 s, respectively, with 1536–4096 transients averaged. A two-dimensional (2D) ^1^H–^13^C heteronuclear correlation (HetCor)^27^ NMR spectrum was measured on cross-linked MCC_3.00_-GA2 to probe the H-bonding, with frequency-switched Lee–Goldburg ^1^H homonuclear decoupling.^28^ A moderate CP time of 0.4 ms was used, and the measurement time was ∼1 day.

## 3 Results and Discussion

### 3.1 Synthesis of Cellulose Glutarate Samples

**Figure 2:**
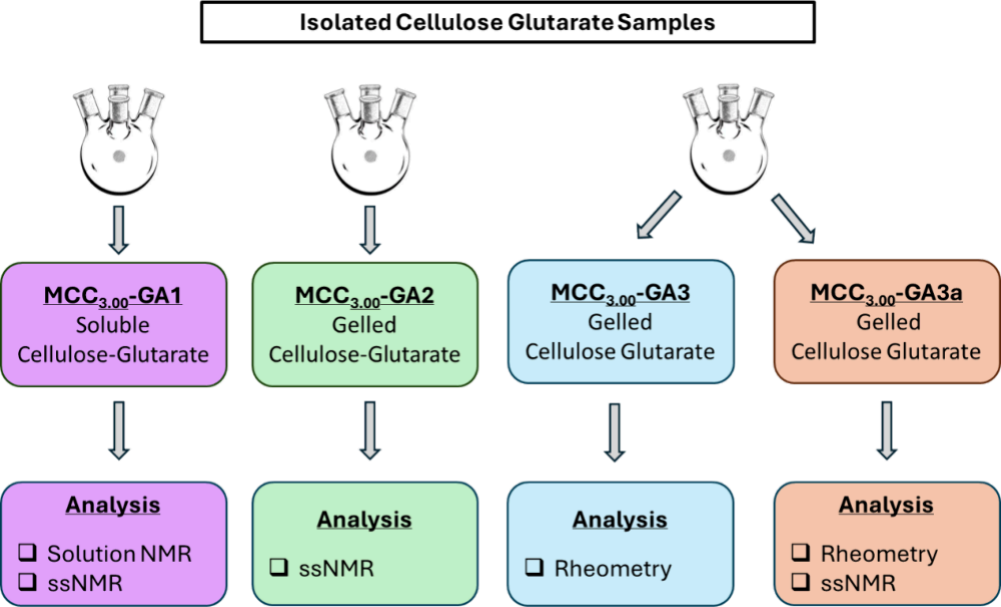
Summary of the different synthesized cellulose glutarate polymer products and their respective analysis (solution NMR, rheometry, and solid-state NMR (ssNMR)).

We previously observed and discussed methods to avoid gelation when reacting cellulose and several different cellulose ester substrates with glutaric anhydride.^9^ We chose cellulose glutarate (MCC_3.00_-GA) as a model system to study gelation phenomena in order to minimize NMR resonance overlap, permitting optimal observation and quantification of phenomena such as hydrogen bonding through dimerization or other associations. For example, the carbonyls of ester substrates, the ester carbonyls of the glutaryl substituted polymer products, and the resulting free carboxyl groups of the diacids all have similar chemical shifts in the ^13^C NMR spectrum, so it was important to minimize the complexity in such spectra. Three different MCC_3.00_-GA samples were purified and freeze-dried prior to solid-state NMR analysis. Figure 2 summarizes the characterization of the polymer samples explored in this work.

**Figure 3:**
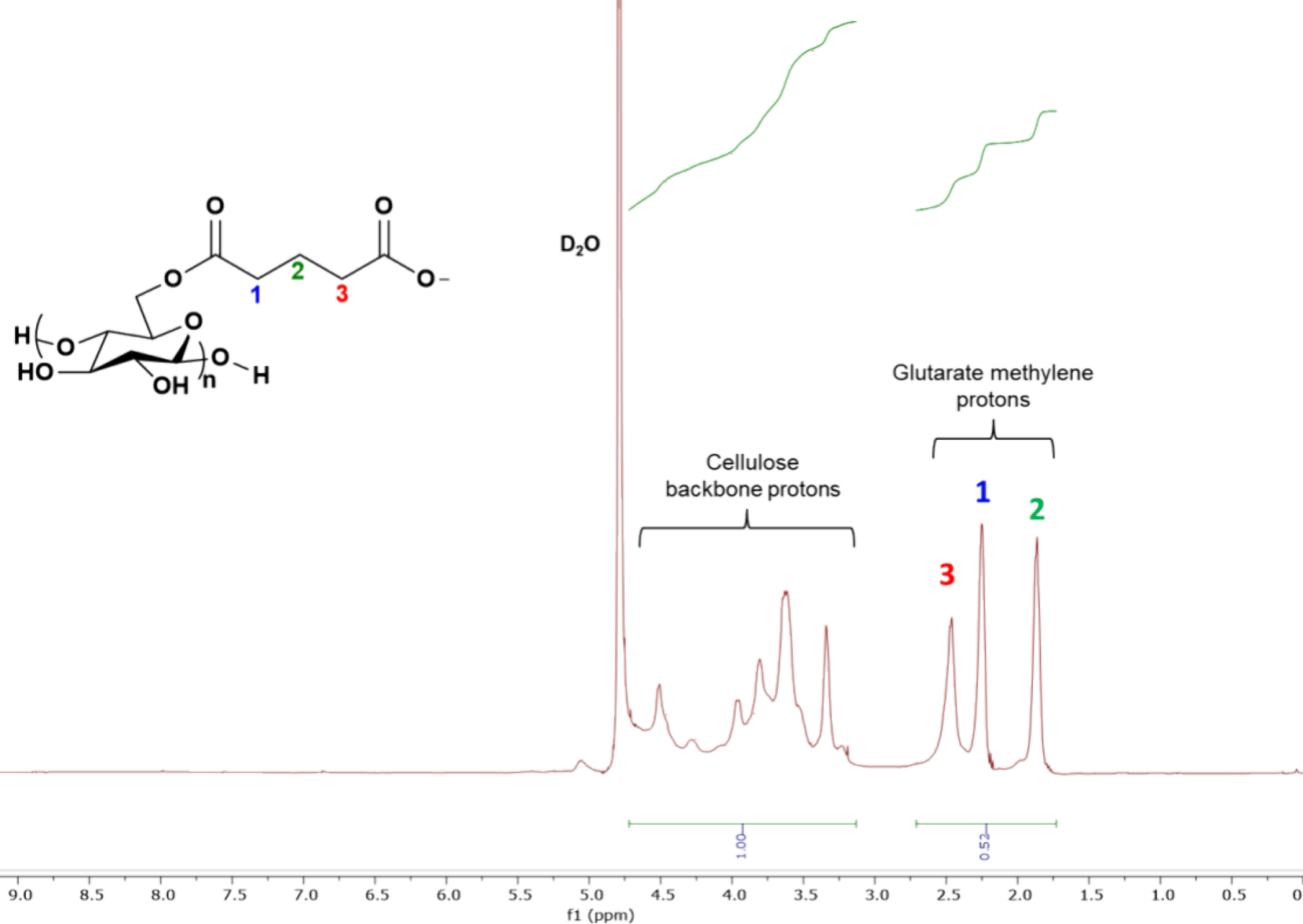
Solution-state ^1^H NMR spectrum (D_2_O) of water-soluble MCC_3.00_-GA1 with DS(GA) of 0.61. The SI provides a method for determining DS (GA) from solution-state ^1^H NMR.

**Scheme 1:**
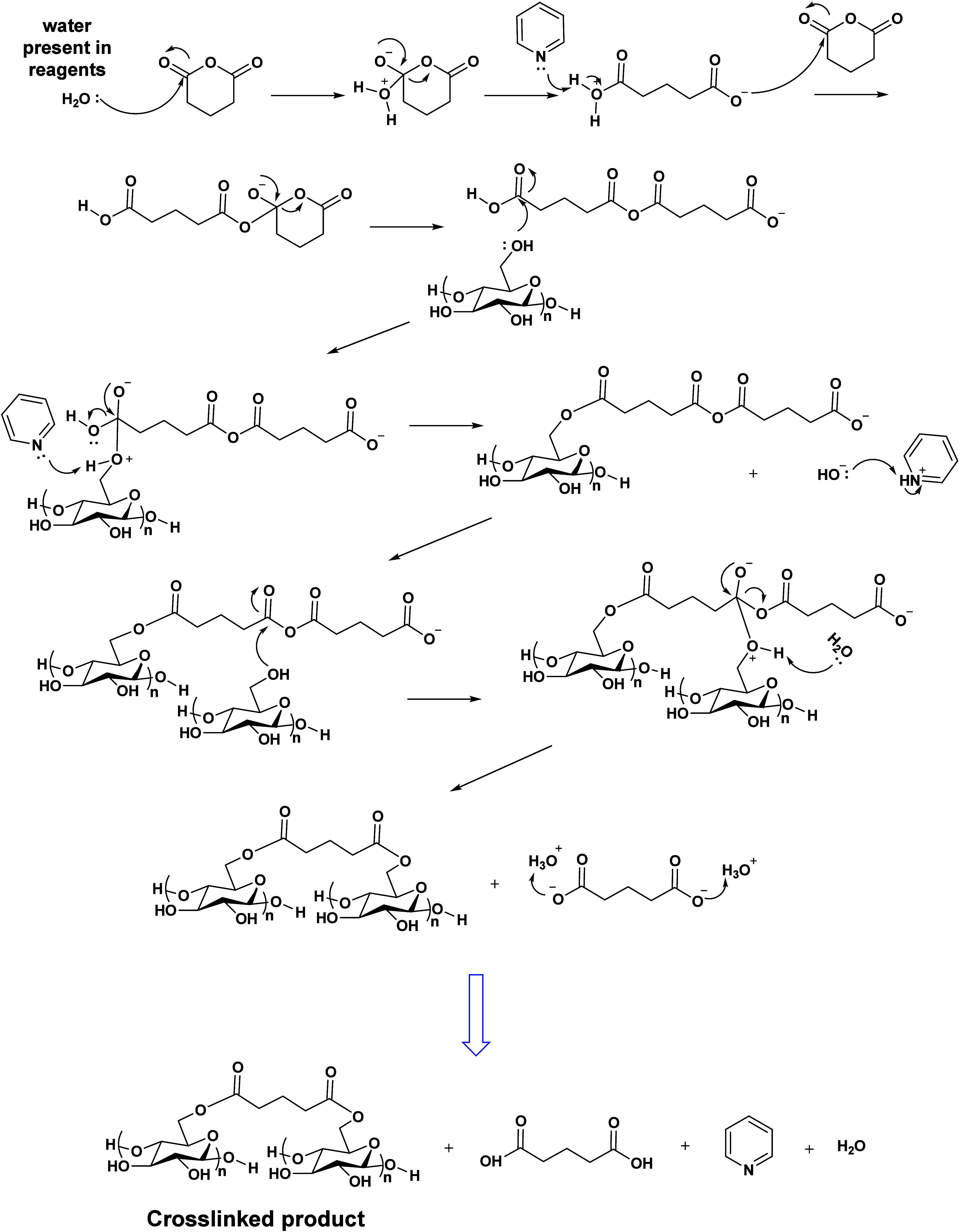
Postulated Crosslinking and Gelation Mechanism during Esterification of Polysaccharides by Cyclic Anhydrides (Mechanism Simplified for Clarity and Not Depicting All Proton Transfer and Mechanistic Steps)

Because the MCC_3.00_-GA samples were either only soluble in D_2_O (Figure 3) or not soluble at all in typical NMR solvents, solution-state NMR was performed on only one representative sample, MCC_3.00_-GA1, yielding a DS(GA) value of ∼0.61. DS calculations from solid-state analysis of this sample evaluated the DS(GA) to be 0.42 ± 0.17 (SI). We attributed the difference in DS(GA) to experimental limitations caused by solubility issues in these materials. The ionized MCC_3.00_-GA1 sample was soluble only in D_2_O. Upon adding trifluoroacetic acid (used to eliminate OH–CH coupling), the polymer precipitated. This impacted the integration of the anhydroglucose CH backbone region, resulting in a slightly inflated calculation of DS(GA) in the solution-state NMR spectrum of MCC_3.00_-GA1 (Figure 3); the DS(GA) from solid-state NMR spectroscopy (Table S1) is likely to be more accurate. This sample and two others, MCC_3.00_-GA2 and MCC_3.00_-GA3a, were evaluated by 1D and 2D solid-state NMR for DS(GA) (SI). As experienced before in nonideal conditions for reactions of cellulose with AA, reactions of cellulose with the six-membered ring GA gelled, typically within 24 h. In our earlier work, to avoid gelation in this system, we purified GA by recrystallization from petroleum ether, dehydrated our reaction solvents, and maintained strictly moisture-free reaction conditions.^9^ Although these reaction conditions significantly decreased gelation in reactions of cellulose alkanoates (cellulose acetate (CA), cellulose acetate propionate (CAP), or cellulose acetate butyrate (CAB)) with GA, gelation was much more difficult to avoid in reactions of GA with cellulose itself. Increasing the DS(OH) appeared to be correlated with faster and more consistent gelation. We initially hypothesized that residual water behaved as a nucleophile and could initiate ring-opening of GA catalyzed by DMAP/pyridine, causing the formation of glutaric acid, which could then initiate GA homopolymerization to form poly(GA) oligomers, much like the poly(AA) oligomers previously observed in reactions of cellulose with AA.^14,15^ Similar oligomers could be initiated from the ω-carboxy-terminated end of one polymer chain (the carboxy group distal from the cellulose main chain) and then could be subject to nucleophilic attack by a hydroxy group of a neighboring cellulose chain, resulting (if the attack is upon an anhydride carbonyl proximal to the cellulose main chain, which one might expect to happen roughly 50% of the time) in covalent cross-linking. Scheme 1 illustrates the proposed GA homopolymerization mechanism. This mechanistic proposal would suggest that the higher the probability of encountering a hydroxy group, the higher the probability of cross-linking, which is consistent with the observation that higher DS(OH) polymers (e.g., cellulose) gel more readily. Similarly, in GA reactions with CAPs differing primarily in DS(OH), reaction with CAP of DS(OH) 0.87 did not cause observable gelation, whereas gelation was observed (unless great care was taken) in reactions with CAP of DS(OH) 1.20.

### 3.2 Rheometry of Cellulose (MCC) Glutarate Solutions to Investigate Gelation

Gelation during reactions of polysaccharides with cyclic anhydrides can impede the generation of useful, discrete polysaccharide products, but the previous literature did not significantly illuminate the nature of the gelation phenomena.^29−32^ In the hypothesized covalent gelation mechanism (Scheme 1), while anhydrides will be reactive with workup components like water or methanol, the oligo(anhydride) chains are hydrophobic and may be part of a gel; thus, migration of the solvolysis reagent to the location of the cross-link may be impeded, and the cross-links may be more durable than one might otherwise expect for a reactive linkage like a carboxylic anhydride. To investigate the gelation mechanism, we performed cellulose esterification using ring-opening of GA and isolated gelled and nongelled material (judged visually) from three separate RO reactions. Gelled samples were analyzed by rheometry and/or quantitative solid-state ^13^C NMR spectroscopy, seeking evidence that would help us distinguish between two gelation hypotheses:

**Hypothesis 1**: Chain extension of the glutarate esters occurs to create reactive oligo(anhydride) substituents that react with hydroxy groups on separate chains to create covalent cross-links.

**Hypothesis 2**: Gels form via physical cross-links.

**Figure 4:**
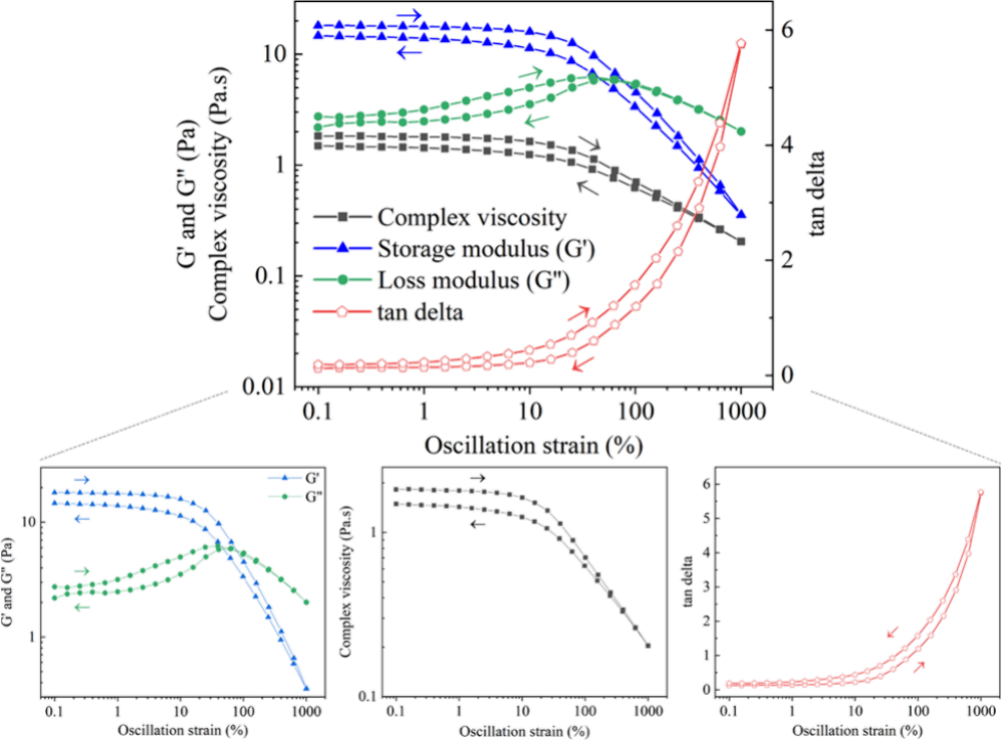
Rheological analyses of purified and reswelled, gelled MCC_3.00_-GA3. Crossover of storage (*G*′) and loss moduli (*G*″) occurs at ca. 80% strain.

If oligo(anhydride) chain extension occurs from the polymer backbone, then the gelation may be covalent. However, if chain extension is not occurring, then the gelation may instead be physical. Rheometry is a valuable tool for providing evidence of the gelation mechanism since gel rheological behavior should be sensitive to the type of cross-linking.^33^ We examined an MCC_3.00_-GA product that had gelled, dividing the sample into two portions. One portion was immediately processed and washed with methanol numerous times to remove DMAc solvent and unreacted small molecule reagents, then reswelled with diH_2_O (MCC_3.00_-GA3). Separately, a crude, gelled sample from the same batch was stored in a glass jar at RT for 1 week (MCC_3.00_-GA3a). For gels formed due to physical cross-links, we would expect an initially higher storage than loss modulus (*G*′ > *G″*) at low deformation, but eventually observe a crossover between moduli (*G*′ = *G″*) under applied stress and elevated strains, indicating breaking of the physical network and transition from solid-like to liquid-like behavior (*G″* > *G*′). However, if cross-linking was covalent, then the gels would be expected to resist deformation and have consistently higher storage than loss modulus (*G′* > *G*″**) under applied stress and with elevated strain values.^34^ Preliminary rheological studies of gelled, purified MCC_3.00_-GA3 were consistent with the hypothesis that the gel displayed physical rather than chemical covalent cross-linking, since under applied shear stress, a crossover between *G*′** and *G*″** occurred, leading to liquid-like behavior (*G″* > *G*′**). Furthermore, with increasing strain and deformation, the gel viscosity decreased, and we observed an increase in tan δ (ratio of loss (*G*″**) to storage modulus (*G′*)). This indicates that gel cross-linking may be caused by physical interactions of the polymer chains, which disentangle, align, and dissipate energy when subjected to increasing force, thereby behaving as a viscous liquid (Figure 4).

Based on these rheometric studies, we then hypothesized that perhaps ion pairing between our catalyst system (DMAP/pyridine) and our carboxylic acid functionality and/or carboxylic acid dimerization (as the DS(COOH) increases over time) was encouraging physical gelation through the formation of extensive hydrogen bonding networks and salt formation between acidic and basic groups (SI).^35−37^ Physical gelation could also result, at least in part, from hydrophobic interactions between the oligo(GA) substituents on different chains if they exist. However, physical gelation does not exclude the possibility of concurrent or subsequent covalent cross-linking through the formation of ester cross-links between different cellulose chains.

**Figure 5:**
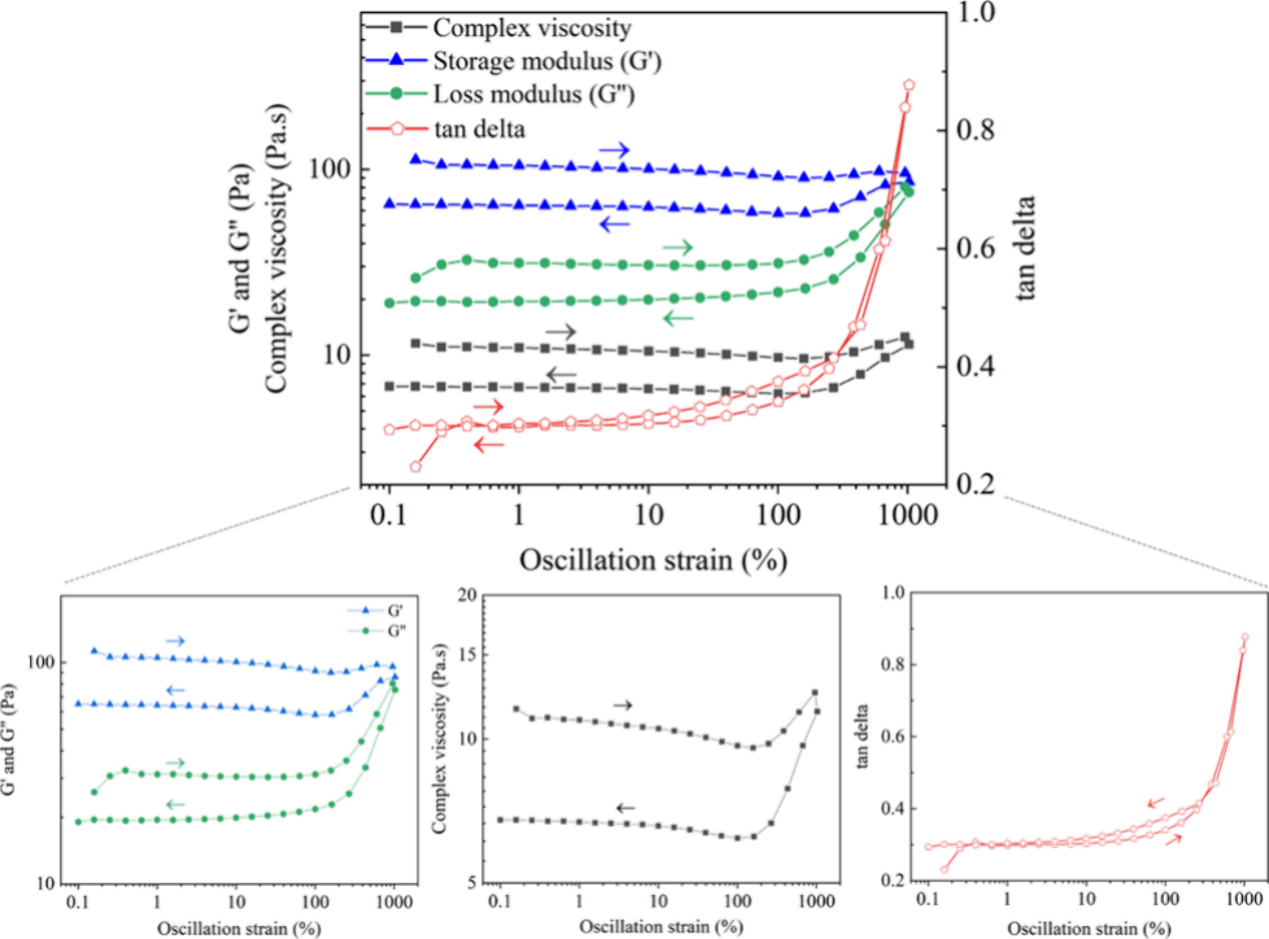
Rheological analyses of gelled, *crude* MCC_3.00_-GA3a. No crossover of storage (*G*′) and loss moduli (*G*″) was observed as a function of increasing strain.

Given sufficient proximity between COOH and OH groups, covalent cross-linking certainly may occur, especially since polymer chain diffusion decreases over time as reaction solution viscosity increases and chain mobility decreases. Interestingly, when we examined the crude sample that was not purified and was kept in the jar for a week (MCC_3.00_-GA3a), rheological analysis more strongly indicated behavior characteristic of chemical covalent cross-linking (Figure 5) relative to the sample that was washed with methanol and purified immediately (Figure 4). The crude, stored sample had no cross-over between *G′* and *G*″ up to 1000% strain, displaying solid-like behavior.

### 3.3 Solid-State NMR Analysis of MCC Glutarate Samples To Investigate Gelation

**Figure 6:**
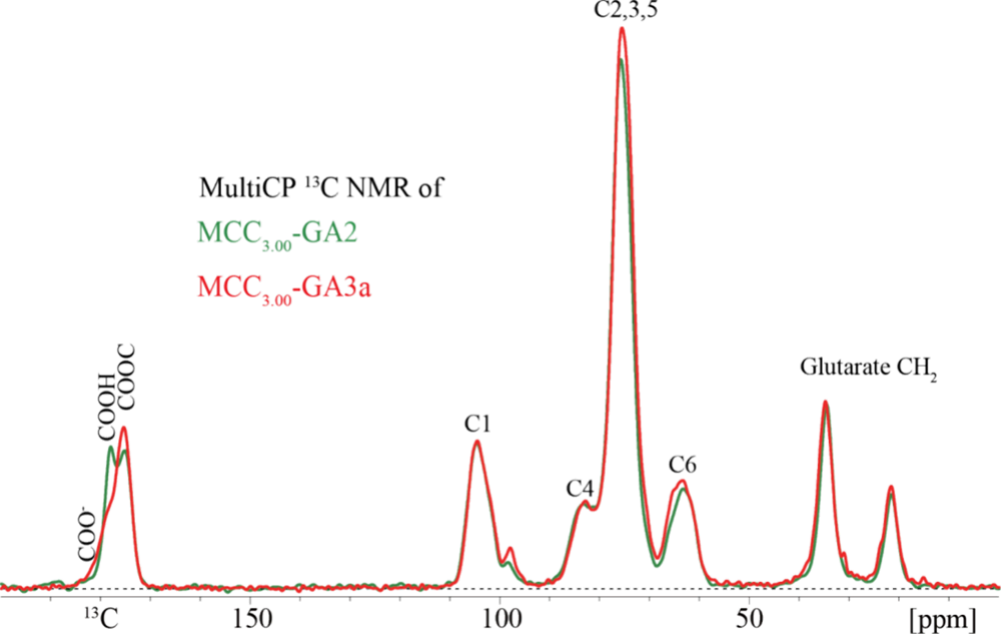
Solid-state ^13^C NMR spectra of gelled MCC_3.00_-GA2 and MCC_3.00_-GA3a samples measured quantitatively by multiCP.

**Figure 7:**
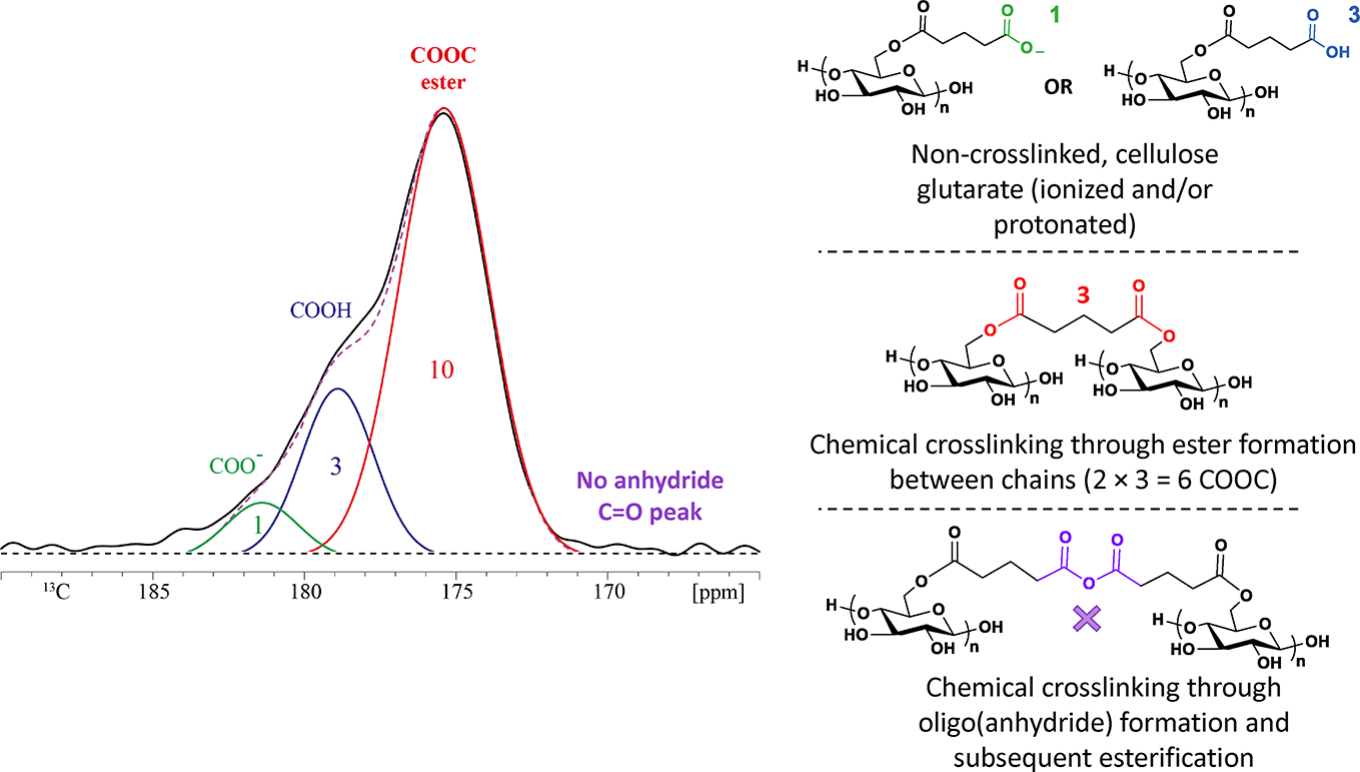
Solid-state ^13^C NMR spectral deconvolution of the COO region of the MCC_3.00_-GA3a sample.

**Figure 8:**
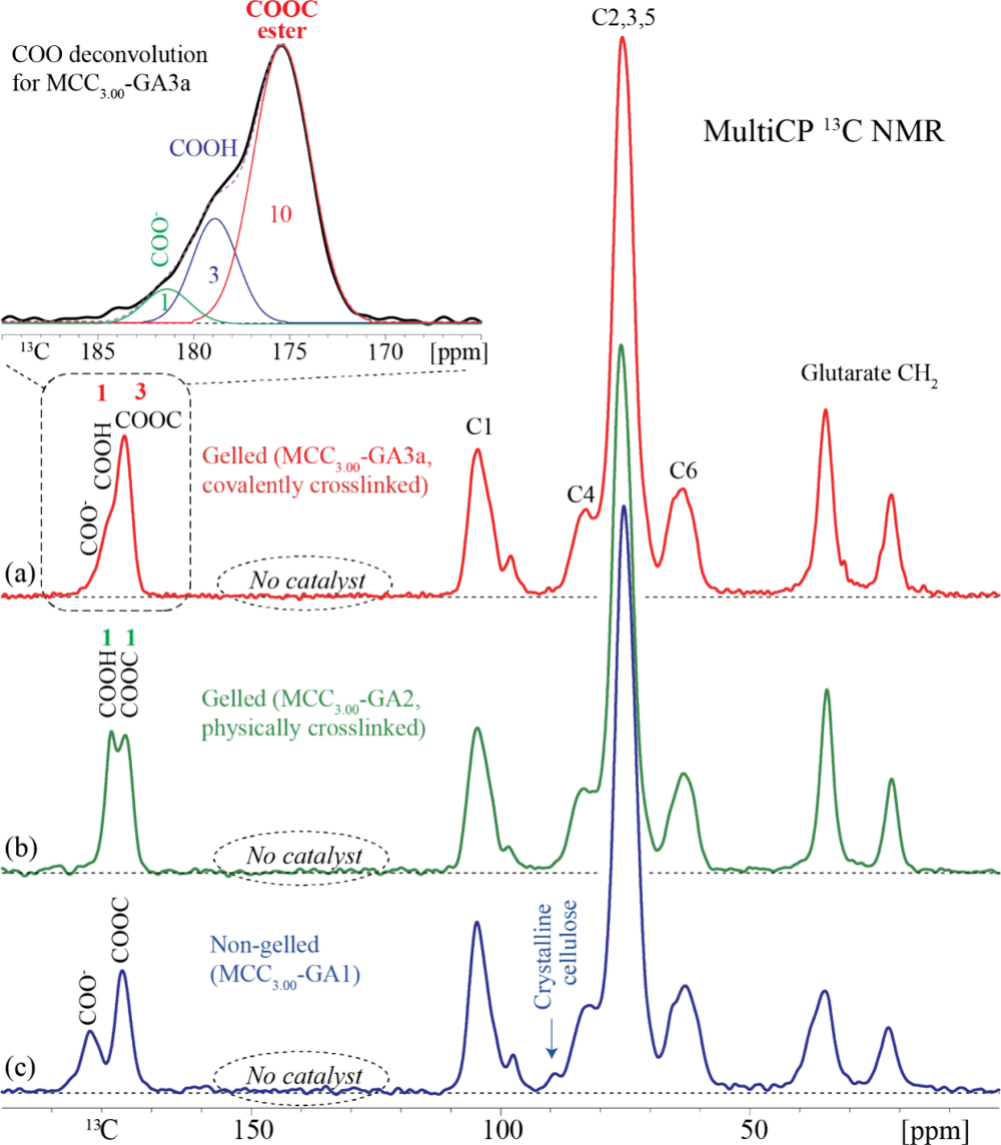
Stacked solid-state ^13^C NMR spectra of (a) gelled (covalently cross-linked, MCC_3.00_-GA3a), (b) gelled (physically cross-linked, MCC_3.00_-GA2) and (c) nongelled (MCC_3.00_-GA1) samples, obtained using quantitative multiCP. COO^–^ and COOH are deprotonated and protonated carboxyl groups, respectively, while COOC refers to the carbonyl from the ester group that is formed upon esterification of cellulose with GA. C1–C6 are the carbons on the anhydroglucose ring. The spectral deconvolution of COO signals in gelled (covalently cross-linked) MCC_3.00_-GA3a is shown as an inset in (a), same as that shown in Figure 7; no anhydride carbonyl signals are observed at ≤171 ppm.

To further probe and quantify these physical versus covalent interactions, we employed solid-state ^13^C NMR spectroscopy to analyze (1) two purified, gelled samples (MCC_3.00_-GA2, MCC_3.00_-GA3a) and (2) a purified sample that had not (visually) gelled (MCC_3.00_-GA1). Gelation was initially qualitatively evaluated based on solubility studies of the samples in pH 6.8 phosphate-buffered saline (described in the Methods section). Solid-state ^13^C NMR spectroscopy provided important insights. First, carboxyl carbon, ester carbonyl, and anhydride carbonyl resonances can be observed between 185 and 162 ppm in ^13^C NMR spectra.^15^ For the covalently cross-linked gelled sample (based on the preliminary rheology results), MCC_3.00_-GA3a, the main COOC ester peak (connecting glutaryl to cellulose hydroxyl) was centered at 175 ppm, while the COOH carbon (corresponding to the terminal COOH of the attached glutaryl moiety) appeared as a shoulder near 179 ppm. A minor amount of carboxylate, COO^–^, was also found to contribute to the foot of the overlapped region at around 182 ppm. No *anhydride* carbonyl carbon resonance was observed where expected, at or below 171 ppm (Figures 6 and 7);^15,38,39^ therefore, our hypothesis that either preformed poly(glutaric anhydride) oligomers and/or oligomerization initiated by a cellulosic hydroxyl occurred was **refuted** for reactions of cellulose with GA. However, the spectrum in Figure 6 provided clear evidence of new ester formation contributing to chemically covalent cross-linking in the MCC_3.00_-GA3a sample (i.e., the crude polymer sample that was stored in a jar for a week). The ratio of carboxyl groups from the glutarate attached to the cellulose backbone, resonating at 179 ppm, relative to the ester carbonyl formed (COOC) between GA and the hydroxyl groups on cellulose, observed at 175 ppm, was approximately 1:3, indicating that the sample is heavily covalently cross-linked (Figures 6, 7, and 8a). That is to say, the observed small ratio tells us conclusively that there are not only Cell-O–CO-glutarate ester carbonyls present but also ester carbonyls resulting from the reaction of glutarate carboxyl termini with OH groups on another cellulose chain, forming an ester cross-link. Simply put, covalent cross-linking converts CO_2_H to ester, reducing the amount of CO_2_H groups and increasing the proportion of esters. The ratio of COOH to ester carbonyls would be 1:1 if no covalent cross-linking had occurred, which was in fact the case for a gelled sample that was *physically* cross-linked, MCC_3.00_-GA2, as shown by the green-line spectra in Figures 6 and 8b. Indeed, the signal areas of the COOH and COOC resonances for the physically cross-linked sample were almost identical. Furthermore, a comparison of the covalently and physically cross-linked samples (MCC_3.00_-GA3a and MCC_3.00_-GA2, respectively) showed substantial differences in the COOC ester region (∼175 ppm) between the two samples. The COOC ester signal of the covalently cross-linked MCC_3.00_-GA3a had a significantly higher intensity relative to the COOC ester peak of the physically cross-linked sample (Figure 6).

Deconvolution of the solid-state ^13^C NMR spectrum of the covalently cross-linked sample, MCC_3.00_-GA3a, revealed that its ratio of COO^–^:COOH:COOC was 1:3:10. This result corresponds to 1/7 of the glutarate termini existing as ionized carboxylic acid (COO^–^) and 3/7 as protonated carboxylic acid (COOH). The absence of anhydride carbonyls means that each carboxyl carbon must be balanced by a glutarate ester linkage to cellulose; that is to say, 4 of the 10 COOC bonds. That leaves 6 other COOC bonds, corresponding to 3 cross-links resulting from esters formed with one cellulose chain on one end of the glutarate entity and another cellulose chain at the other end of the glutarate entity (so 2 × 3 = the 6 COOC observed) (Figure 7).

Further analysis of the quantitative ^13^C NMR spectra for all three (soluble, physically, and covalently cross-linked) samples also showed no evidence of residual DMAP/pyridine catalysts since there were no aromatic resonances observed in the ^13^C NMR spectrum (110–150 ppm) above the detection limit of 2% C (Figure 8). Therefore, there was no evidence of salt formation or ion pairing occurring between the carboxyl functional groups and the amine catalysts.

**Figure 9:**
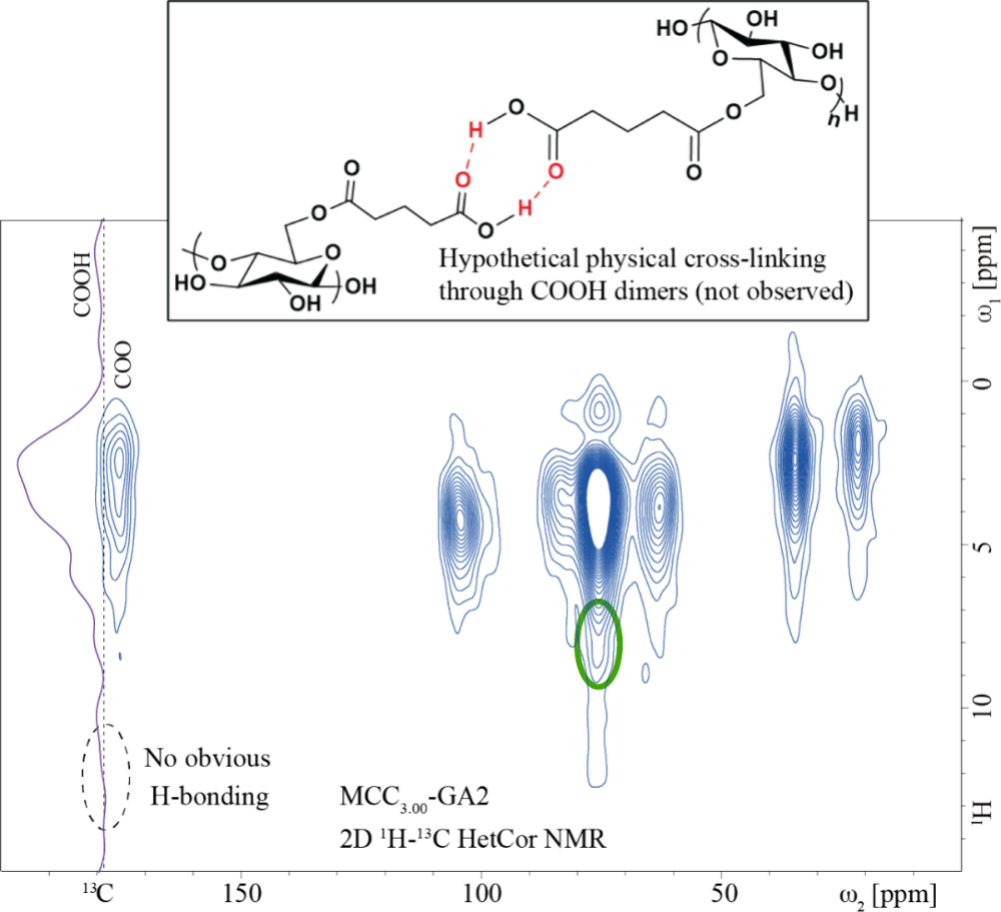
Solid-state 2D ^1^H–^13^C HetCor NMR spectrum of physically gelled MCC_3.00_-GA2 measured at 14 kHz MAS, superimposed (near the left edge, purple trace) with a vertical 1D ^1^H cross-section at the COOH resonance position.

To evaluate whether carboxylic acid dimerization through H-bonding between glutarates was present and contributed to gelation, we employed 2D ^1^H–^13^C HetCor NMR analysis. If there were H-bonds present because of carboxylic acid dimerization, then we would expect a downfield shift in the corresponding carboxylic acid proton cross peaks.^40^ As shown in Figure 9, there is no evidence of H-bonding resulting from carboxylic acid dimerization. The vertical ^1^H cross section at the COOH resonance position confirms that there are no new ^1^H resonances corresponding to the downfield shift of COOH, typically >10 ppm, which would result from deshielding effects and is an indication of H-bonding.^40^ Generally, the COOH resonance is weak here, indicating its poor cross-polarization due to fast large-amplitude motions of free COOH groups at the end of flexible side groups without the constraints that would be imposed by carboxylic acid dimerization. The high mobility of the COOH group is also confirmed by its relatively fast ^13^C spin–lattice relaxation.

Therefore, it is likely that for the physically cross-linked MCC_3.00_-GA2 sample, the gels predominantly form due to other physical interactions, such as hydrophobic interactions, stacking between the polymer chains, chain entanglements, and H-bonding between free hydroxyl groups on the cellulose backbone. The 2D ^1^H–^13^C HetCor NMR data do suggest that there is H-bonding between the free hydroxyl groups. The cross peak supporting this conclusion is circled green in Figure 9.

Overall, the quantitative solid-state NMR results were consistent with those from the rheological experiments. Further, there was no evidence for oligo(anhydride) chain extension in either physically or covalently cross-linked samples, and no evidence of anhydride cross-links as determined by solid-state NMR, therefore refuting ***Hypothesis 1***. Rheological analyses of the crude MCC_3.00_-GA3a sample showed that over time covalent cross-linking can occur if an unprocessed, gelled sample is stored long enough even at RT, demonstrated by the change in the rheology curves in Figure 5 when compared with Figure 4 (immediately purified polymer, MCC_3.00_-GA3, from the same reaction batch). After the rheometry results revealed that MCC_3.00_-GA3a exhibited curves consistent with covalent cross-linking behavior, we performed solid-state NMR analysis of MCC_3.00_-GA3a. We found that, indeed, this sample exhibited significant ester cross-linking between neighboring cellulose chains facilitated by glutarate acting as a homobifunctional cross-linker. These spectroscopic results corroborated what was observed in the rheometric analysis of this sample.

These results provide considerable insight into the gelation mechanisms between cellulose and cyclic anhydrides. The reactivity of the cyclic anhydride depends significantly on ring size, and this would be expected to impact the gelation mechanism. Regarding reactivity, it is useful to compare the strain energies of 5- (succinic) and 6-membered (glutaric) cyclic anhydrides previously determined through combustion analysis and reported as follows: 3.3 kJ/mol for succinic anhydride and 19.4 kJ/mol for glutaric anhydride.^41^ The GA 6-membered ring is significantly more strained, and therefore more prone to ring-opening (due to the 16.1 kJ/mol difference) than the 5-membered SA. Although the strain energy of adipic anhydride has not been reported, the ring torsion and destabilizing angle distortions from seven-membered rings are well-documented in other cyclic ketone systems such as lactone derivatives.^42^ Computational modeling of succinic, glutaric, and adipic anhydride evaluated the difference in energy between their closed (cyclic anhydride) and opened (linear diacid) forms using DMAc as the implicit solvent and water as the proton donor (Table S3). The computed output energies were as follows: 27.8, 40.0, and 79.5 kJ/mol for SA, GA, and AA, respectively. The modeling results showed a trend similar to that in the previously reported combustion analysis results for ring strain. Increasing the ring size of the cyclic anhydride yields significant energy differences between the three different rings, where the energy difference is 12.2 kJ/mol between SA and GA, 39.5 kJ/mol between GA and AA, and 51.7 kJ/mol between SA and AA, thereby impacting the reactivity of the different cyclic anhydrides.

The spontaneous ring-opening of adipic anhydride that we observed, consistent with previous literature reports and the modeling experiments, results from the fact that it is significantly more reactive than either succinic or glutaric anhydrides. This is partly due to the planar conformation of the rigid C–O–C=O–C group that is present within cyclic anhydride systems and imparts torsional strain and greater bond distortion to the rest of the ring.^41,43,44^ Each addition of a new carbon in the ring causes greater bond distortion and increasingly favors ring-opening, even in the absence of a catalyst. The significant strain energy differences between the three rings may help explain why we observed an increased frequency in, and decreasing time to, cross-linking and gelation with increasing ring size. The type of cross-linking mechanism that prevailed also changed with the type of ring and corresponding strain value. We rarely observed gelation with succinic anhydride but observed gelation much more consistently with glutaric anhydride (gelation starting out as physical and turning to chemical covalent over time with no observed glutarate oligomerization).

Meanwhile, reactions with adipic anhydride^14,15^ showed that chemical covalent cross-linking was the dominant cross-linking form in these reactions, which proceeded through a mechanism involving rapid and extensive oligomerization of adipic anhydride in solution and/or through chain extension from the main polysaccharide chain, followed by extensive chemical covalent cross-linking by these poly(adipic anhydride) oligomers with neighboring hydroxy groups on other polysaccharide chains.^14,15^

## 4 Conclusions

Upon reacting microcrystalline cellulose with six-membered cyclic glutaric anhydride, we consistently observed gelation. Although gelation has been observed with 7-membered adipic anhydride, the phenomenon has not been described in detail in the literature, and we have found no literature reports investigating the gelation mechanism. Therefore, we used cellulose glutarate as a model system to understand the mechanisms involved in gelation during cellulose-cyclic anhydride ring-opening esterification.

We explored the covalent vs physical gelation hypotheses described above through rheometry and solid-state ^13^C NMR spectroscopy. The results showed that gelation occurs predominantly through physical interactions rather than covalent cross-linking; however, covalent cross-linking by ester bonds between carboxyl termini of glutarate substituents and OH groups of separate cellulose glutarate chains, *without* any anhydride (oligo(GA)) formation detected by solid-state ^13^C NMR spectroscopy, is observed if unprocessed samples are left for extended periods of time, even at room temperature. For smaller and less reactive cyclic anhydrides (e.g., succinic), gelation was less common, although it has been mentioned in the literature and observed by us as well. This may be due to less favorable self-associations between different polymer chains (ω-carboxyalkanoate chains are shorter and may have weaker van der Waals interactions). It may also be significant that the shorter ω-carboxyalkanoates would need to overcome larger steric interactions between chains (a closer approach) in order to form ester bonds with hydroxyls on those chains. All of these characteristics may reduce the tendency of succinic anhydride systems to gel physically and/or covalently relative to more reactive systems like glutaric anhydride and adipic anhydride.

When we move to the more reactive 6-membered ring, glutaric anhydride, we observe consistent gelation, specifically in cellulosic polymers with high DS(OH), cellulose itself being the most extreme case. However, gelation occurs mostly through extensive hydrophobic interactions, as the preference for polymer self-association increases with increasing DS(GA). We also observed indications of H-bonding between hydroxyls spectroscopically, with the potential for subsequent covalent cross-linking as samples age, even at RT. In contrast, when we carried out cellulose esterification with the highly reactive seven-membered AA in past work, evidence for AA homopolymerization resulting in poly(AA) was observed through the presence of proton peaks in solution-state ^1^H NMR, and further evidence for cross-linking was observed through solid-state NMR spectra and FTIR analysis.^14,15^ These poly(AA) oligomers either formed in situ prior to reacting with cellulose, and/or through oligomerization initiated by cellulosic hydroxyls, leading to extensive and consistent *covalent* cross-linking. We expect that these mechanistic insights will prove valuable for those carrying out ring-opening reactions of polysaccharides and their derivatives with cyclic anhydrides and will also help investigators understand the behavior of some of these products upon storage.

## Supporting Information Available

Solid-state ^13^C NMR spectra of gelled and nongelled MCC_3.00_-GA (cellulose glutarate) obtained quantitatively, followed by the analysis of the solid-state ^13^C NMR and solution ^1^H NMR spectra of three MCC_3.00_-GA samples; solid-state ^13^C multiCP NMR spectral integrals of three cellulose glutarate (MCC_3.00_-GA) samples; schematic illustrating protonated pyridine and/or DMAP interacting with cellulose glutarate carboxylates to form salts in situ; Hartree energy value outputs for cyclic and linear anhydrides using computational modeling; and calculated energy difference values (in kcal/mol) between cyclic and linear anhydride forms using computational modeling (PDF)
