# Supplementary material for: Gelation during Ring-Opening Reactions of Cellulosics with Cyclic Anhydrides: Phenomena and Mechanisms
Source: Biomacromolecules. 2024 Nov 21;25(12):7777–87. doi: 10.1021/acs.biomac.4c01081 (PMC11632662; doi:10.1021/acs.biomac.4c01081)
Supplement: Supplementary file 1 — bm4c01081_si_001.pdf [file bm4c01081_si_001.pdf]

Supporting Information

Gelation During Ring-Opening Reactions of Cellulosics with Cyclic Anhydrides: Phenomena and Mechanisms

Stella P. Petrova*, Zhaoxi Zheng, Daniel Alves Heinze, Valerie Vaissier Welborn, Michael J. Bortner, Klaus Schmidt-Rohr, Kevin J. Edgar

Corresponding author e-mail: stellap@vt.edu

Additional quantitative solid-state NMR and analysis

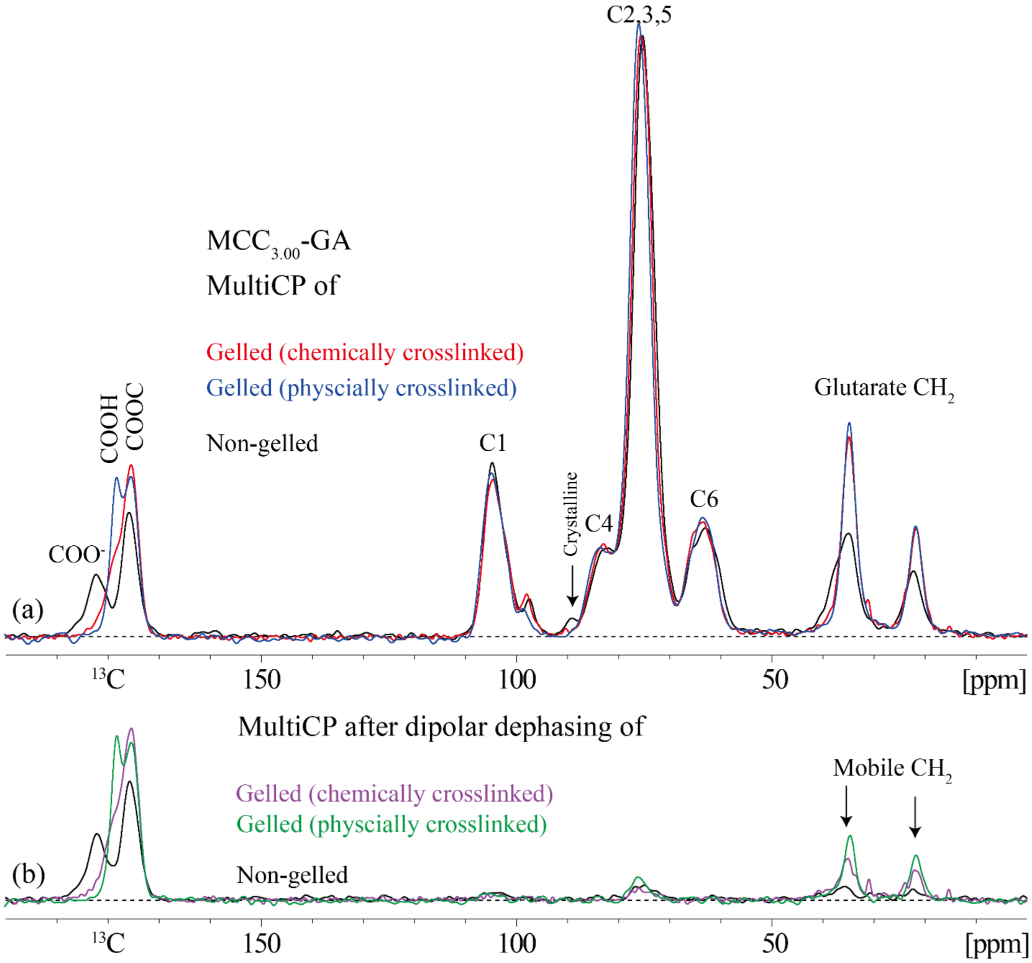

Figure S1. Solid-state ^{13}C NMR spectra of gelled and non-gelled $MCC_{3.00}$-GA obtained quantitatively by (a) multiCP and (b) multiCP after dipolar dephasing, which shows carbons that are not bonded to hydrogen or mobile.

Through analysis of the solid-state ^{13}C NMR and solution ^{1}H NMR spectra, the following mole fractions were determined and quantified in three $MCC_{3.00}$-GA samples:

1. A minor amount of crystalline cellulose was found in the uncrosslinked sample (~5% vs 2% in the crosslinked one), as indicated by the crystalline C4 peak near 89 ppm.

2. Three types of COO groups were found:

   COO^- and COOC in the non-gelled $MCC_{3.00}$-GA1;

   COOH, and COOC in the gelled physically crosslinked, $MCC_{3.00}$-GA2;

   COO^-, COOH, and COOC in the gelled covalently crosslinked, $MCC_{3.00}$-GA3a.

3. The mole ratio of glutarate to cellulose (calculated based on COO to C1 integrals, see Table S1):

   ~0.42 in the non-gelled $MCC_{3.00}$-GA1;

   ~0.52 in the gelled physically crosslinked $MCC_{3.00}$-GA2;

   ~0.49 in the gelled covalently crosslinked $MCC_{3.00}$-GA3a.

4. The degree of substitution (**per cellulose ring**) was calculated to be:

   0.42 ± 0.17 in non-gelled $MCC_{3.00}$-GA1;

   0.52 ± 0.16 in the gelled physically crosslinked $MCC_{3.00}$-GA2;

   $0.70^{a} \pm 0.07$ in the gelled covalently crosslinked $MCC_{3.00}$-GA3a.

5. DS calculation of non-gelled ($MCC_{3.00}$-GA1) based on solution ^1H NMR data (see Figure 3):

   The total cellulose OCH_n backbone integral (from 5+2 protons) is 1, so the mole fraction of cellulose units = 1/7 = 0.143

   The total glutarate CH_2 signal (from 3 methylene carbons × 2 protons) is 0.52, so mole fraction of glutarate units = 0.52/6 = 0.087

   DS = 0.087/0.143 = 0.61

Table S1. ^{13}C multiCP NMR spectral integrals of three cellulose glutarate ($MCC_{3.00}$-GA) samples. Degree of substitution (DS) values were calculated by dividing COO integrals by two, with error margins obtained based on both COO and less reliable C=O-CH_2 integrals. Cellu = cellulose backbone.

**Sample**	**COO**	**Cellu C1**	**Cellu C2-5**	**C=O-CH_2**	**C-CH_2**	**DS**
**$MCC_{3.00}$-GA1**	0.84	1	3.93	0.67	0.35	0.42 ± 0.17
**$MCC_{3.00}$-GA2**	1.04	1	4.0	0.88	0.43	0.52 ± 0.16
**$MCC_{3.00}$-GA3a**	0.98	1	3.94	0.91	0.45	0.7* ± 0.07

*For the $MCC_{3.00}$-GA3a sample, extra substitution from double esters was taken into account. The DS was calculated as 0.98/2 + 0.98×3/14 = 0.7

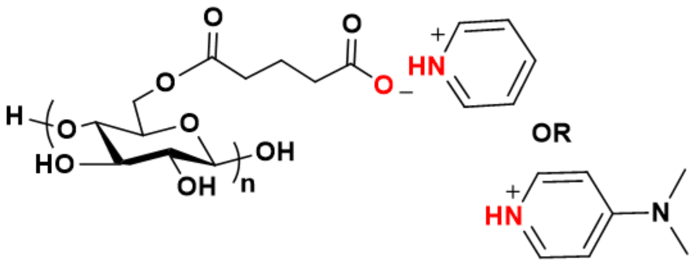

Figure S2. Schematic illustrating protonated pyridine and/or DMAP interacting with cellulose glutarate carboxylates to form salts *in situ*.

Computational modeling results

Table S2. Hartree energy value outputs for cyclic and linear anhydrides. To keep the number of atoms consistent between cyclic and linear forms, modeling was performed with the anhydride and with water molecules as the proton source. The implicit solvent, DMAc, used for the modeling studies is listed in column A. Calculations are also performed in a vacuum-simulated environment to obtain baseline Hartree values. Columns B and D pertain to the cyclic anhydride by itself (B) and cyclic anhydride with water as a proton donor (D). Columns C and E pertain to the water molecule by itself and the linear acid form of the cyclic anhydride, respectively.

	A	B	C	D	E
**1**	**Hartrees**	**Succinic**			
**2**	**Implicit Solvent**	**Anhydride**	**Water**	**Anhydride with water**	**Acid carboxyl**
**3**	Vacuum	-380.550	-76.440	-456.996	-457.000
**4**	DMAc	-380.563	-76.446	-457.014	-457.020
**5**					
**6**		**Glutaric**			
**7**	**Implicit Solvent**	**Anhydride**	**Water**	**Anhydride with water**	**Acid carboxyl**
**8**	Vacuum	-419.861	-76.440	-496.309	-496.313
**9**	DMAc	-419.876	-76.446	-496.328	-496.337
**10**					
**11**		**Adipic**			
**12**	**Implicit Solvent**	**Anhydride**	**Water**	**Anhydride with water**	**Acid carboxyl**
**13**	Vacuum	-459.165	-76.440	-535.620	-535.632
**14**	DMAc	-459.178	-76.446	-535.630	-535.655
**15**					
**16**	**Atom count**	**17 atoms**	**3 atoms**	**20 atoms**	**20 atoms**

Table S3. Calculated energy difference values (in kcal/mol) between cyclic and linear anhydride forms optimized with water to keep the number of atoms consistent between cyclic and linear forms as per Table S1. Calculations were performed using the Hartree values in Table S1. The implicit "solvents" used are listed on the left column. The calculations were performed by adding the DMAc values in column B and column C, then subtracting the value in column E. To convert from Hartree to kcal/mol, the resulting value was multiplying by 627.509. An example calculation looks like the following: (Column B4 + Column C4 – Column E4) *627.509 = 6.655 kcal/mol for succinic anhydride.

	**Succinic**
**Implicit Solvent**	**Anhydride + water - carboxylic (kcal/mol)**
Vacuum	6.353
DMAc	6.655
	**Glutaric**
	**Anhydride + water - carboxylic (kcal/mol)**
Vacuum	7.253
DMAc	9.560
	**Adipic**
	**Anhydride + water - carboxylic (kcal/mol)**
Vacuum	16.595
DMAc	19.002

The DMAc highlighted energy values correspond to the values used in the manuscript. To convert from **kcal/mol** to **kJ/mol**, the highlighted energy values need to be multiplied by **4.184 kJ/mol**.
